# Efficacy assessments of EL219, a next-generation polyene antifungal, in immunosuppressed mice infected with drug-sensitive and drug-resistant *Aspergillus* isolates

**DOI:** 10.1128/aac.01400-25

**Published:** 2025-12-05

**Authors:** Eman G. Youssef, Teclegiorgis Gebremariam, Yiyou Gu, Hoja Patterson, Sondus Alkhazraji, Tasneem Elsayed, Nathan P. Wiederhold, Ashraf S. Ibrahim

**Affiliations:** 1 Division of Infectious Diseases, The Lundquist Institute for Biomedical Innovation at Harbor-University of California Los Anegeles (UCLA) Medical Center 5116, Torrance, California, USA; 2 University of Texas Health Science Center at San Antonio 14742, San Antonio, Texas, USA; 3 David Geffen School of Medicine at UCLA 12222 , Los Angeles, California, USA; University of Iowa, Iowa City, Iowa, USA

**Keywords:** EL219, SF001, polyene, antifungal agents, *Aspergillus*, murine, infection model, liposomal amphotericin B

## Abstract

Invasive pulmonary aspergillosis (IPA), mainly caused by *A. fumigatus,* remains a life-threatening fungal infection, with rising reports of infections caused by resistant species such as *Aspergillus lentulus* and *Aspergillus calidoustus*. EL219 (formerly known as SF001) is a novel, next-generation polyene with enhanced ergosterol selectivity and reduced nephrotoxicity. We evaluated the efficacy of EL219 compared to liposomal amphotericin B (LAMB) *in vitro* using the Clinical Laboratory and Standards Institute M38 methodology and in immunosuppressed murine models of IPA caused by *A. fumigatus*, *A. lentulus*, and *A. calidoustus*. Immunosuppressed ICR mice were infected via inhalation (*A. fumigatus*) or via intratracheal instillation (*A. lentulus* and *A. calidoustus*), then treated intravenously once daily with placebo, EL219 (0.3, 1.5, 7.5, and 30 mg/kg), or LAMB (5 mg/kg) starting 16 h post-infection. Treatment lasted 4 days (*A. fumigatus* and *A. lentulus*) or 7 days (*A. calidoustus*). Survival through Day 21 and lung fungal burden at Day 4 were assessed. EL219 showed enhanced *in vitro* activity against *A. lentulus* and *A. calidoustus* compared to LAMB. EL219 significantly improved survival in a dose-dependent manner, with 30 mg/kg outperforming LAMB. EL219 also resulted in >5-log fungal burden reductions in *A. fumigatus*-infected lungs. EL219 demonstrated broad-spectrum efficacy, improved survival, and reduced lung fungal burden, supporting its potential as a novel therapy for IPA.

## INTRODUCTION

Invasive pulmonary aspergillosis (IPA) is a life-threatening fungal infection that primarily affects immunocompromised individuals, including those undergoing chemotherapy, hematopoietic stem cell transplantation, or receiving prolonged corticosteroid therapy (1–3). Over the past two decades, the incidence of IPA has increased significantly, largely due to the expanding population of patients with immune dysfunction and the widespread use of immunosuppressive therapies (4, 5).

*Aspergillus fumigatus* is the most commonly isolated species from patients with IPA, accounting for over 90% of cases in immunocompromised patients (6, 7). However, emerging non-*fumigatus* species such as *A. lentulus* and *A. calidoustus* are increasingly being reported in clinical settings, particularly in patients with hematological malignancies and transplant recipients (8, 9). Cryptic species, such as *A. lentulus,* are often misidentified as *A. fumigatus*, and both *A. lentulus* and *A. calidoustus* can be resistant to multiple antifungals, including triazoles and amphotericin B formulations, complicating treatment and contributing to poor clinical outcomes (8, 10). Notably, infections caused by *A. lentulus* and *A. calidoustus* are associated with higher mortality rates compared to *A. fumigatus*, due to both diagnostic challenges and limited treatment options (3, 7, 11).

Current antifungal therapies for IPA primarily include triazole agents such as voriconazole, posaconazole, and isavuconazole, as well as liposomal amphotericin B (LAMB) (12, 13). While these agents remain the standard of care, they are often associated with limitations. Amphotericin B and its lipid formulations retain nephrotoxicity due to their non-specific binding to human cholesterol (14, 15), while azoles show variable efficacy due to increasing resistance, especially in non-*fumigatus* strains (11, 16). In patients with hematologic malignancies and prolonged neutropenia, antifungal treatment failure is common, with reported IPA-related mortality reaching 50–70% and even higher in disseminated cases (17, 18). These clinical challenges highlight the urgent need for novel antifungal agents with improved safety and broad-spectrum activity, particularly against resistant and emerging *Aspergillus* species.

EL219 (formerly known as SF001 and AM-2-19) is a next-generation, rationally designed polyene antifungal with high specificity for fungal ergosterol and reduced affinity for human cholesterol, offering a significantly improved toxicity profile (19). It is formulated micellarly in a 1:3 molar ratio with DSG-PEG2000. In preclinical studies, EL219 demonstrated potent broad-spectrum *in vitro* fungicidal activity against a range of clinically important fungal pathogens, including *Candida*, *Aspergillus*, *Cryptococcus*, *Histoplasma*, *Coccidioides*, *Blastomyces*, *Talaromyces*, and members of the Mucorales order (19, 20). Moreover, EL219 has shown promising *in vivo* efficacy in murine models of invasive candidiasis, mucormycosis, fusariosis, and disseminated aspergillosis, with a significant reduction in fungal burden in tissues (19, 21, 22).

In this study, we build upon previous findings by evaluating the efficacy of EL219 in established murine models of IPA caused by *A. fumigatus*, *A. lentulus*, and *A. calidoustus*. These clinical isolates were selected for their multidrug-resistant profiles. We assessed survival, lung fungal burden, and histopathological outcomes in immunosuppressed mice treated with EL219 compared to those treated with LAMB. Our goal was to determine whether EL219 could offer superior protection against these challenging *Aspergillus* infections and to further support its potential as a broad-spectrum antifungal agent.

## RESULTS

### EL219 overcomes *in vitro* LAMB resistance in *A. lentulus* and *A. calidoustus* clinical isolates

EL219 *in vitro* activity was evaluated and compared to LAMB and AMB against different clinical isolates of *A. fumigatus*, *A. lentulus*, or *A. calidoustus* using the DSG-PEG2000 micellar *in vivo* formulation in accordance with the Clinical Laboratory and Standards Institute (CLSI) M38 broth microdilution methodology (23). Minimum inhibitory concentrations (MICs) were determined by visually assessing 100% growth inhibition after 48 h of incubation at 35°C. EL219 exhibited comparable MIC values for LAMB against *A. fumigatus* MICs ranging from 0.5 to 1.0 µg/mL versus 0.06–0.5 for LAMB (*n* = 6 isolates). In contrast, EL219 exhibited notably lower MIC values against LAMB-resistant species of *A. lentulus* (*n* = 6), and *A. calidoustus* (*n* = 6). Specifically, the range of MIC values for EL219 against *A. lentulus* was 2.0–4.0 and 0.5–1.0 µg/mL for *A. calidustious* versus LAMB for *A. lentulus* of 4 to >16 and >16 µg/mL for *A. calidustious* (Table 1). The favorable EL219 MICs compared to LAMB at 100% inhibition were similar to those obtained for AMB at this endpoint (range: 0.25–4 µg/mL for all strains tested).

**TABLE 1:** EL219, LAMB, and AMB MIC values for *Aspergillus* strains evaluated

*Aspergillus* species	Strain	MIC (µg/mL)		
		EL219	LAMB	AMB
*A. fumigatus*	Af293^*a*^	0.5	0.06	1
	Af-1	1	0.125	0.25
	Af-2	0.5	0.125	2
	Af-3	0.5	0.06	0.5
	Af-4	0.5	0.125	1
	Af-5	0.5	0.5	1
*A. lentulus*	Al-1^*a*^	2	>16	4
	Al-2	4	>16	ND^*b*^
	Al-3	4	>16	2
	Al-4	2	4	1
	Al-5	2	>16	ND
	Al-6	2	>16	4
*A. calidoustus*	Ac-1	1	>16	2
	Ac-2	1	>16	2
	Ac-3	1	>16	ND
	Ac-4	0.5	>16	1
	Ac-5	1	>16	2
	Ac-6^*a*^	1	>16	2

^*a*^ Used strains in the animal studies.

^*b*^ ND, not determined.

### EL219 shows superior efficacy to LAMB in immunosuppressed mice infected with *A. fumigatus*

**Fig 1:**
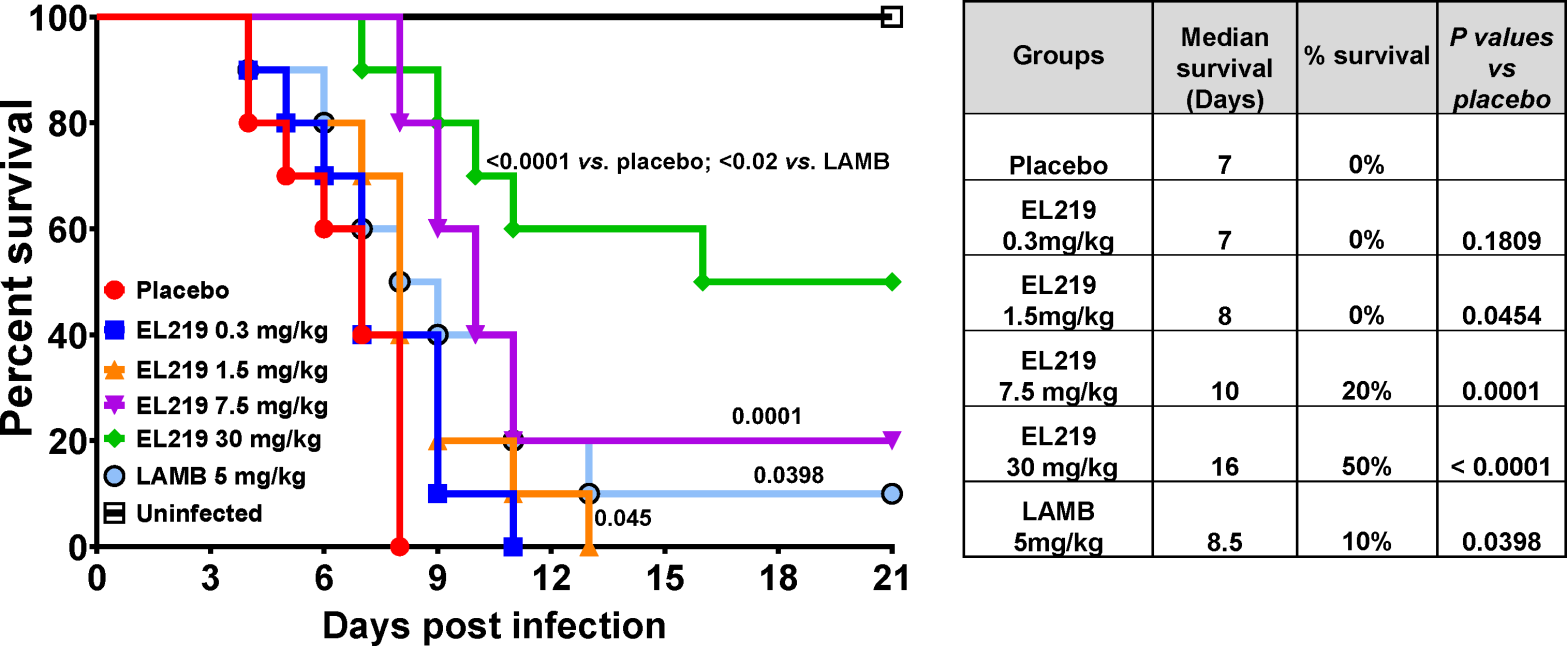
Survival of immunosuppressed mice infected with *A. fumigatus* Af293 and treated with either EL219 or LAMB. Mice (*n* = 10/group) were rendered immunosuppressed and infected via inhalation with an inoculum of 2.4×10^4^ conidia. Treatment began 16 h post-infection and continued once daily for four consecutive days. EL219 at doses of 1.5, 7.5, or 30 mg/kg prolonged median survival compared to placebo. Doses of 7.5 or 30 mg/kg EL219, as well as LAMB at 5 mg/kg, improved overall survival compared to placebo (20%, 50%, 10%, and 0% survival for 7.5, 30 mg/kg EL219, LAMB, and placebo, respectively). Numbers on the figure represent *P* values compared to placebo or as stated in the figure. The accompanying table presents median survival times and overall survival percentages through Day 21 post-infection.

To evaluate the efficacy of EL219 in treating IPA, immunosuppressed mice were infected with *A. fumigatus* Af293 via an inhalation chamber and treated with EL219 at doses of 0.3, 1.5, 7.5, or 30 mg/kg once daily for four consecutive days, starting 16 h post-infection. LAMB at 5 mg/kg was used as the comparator. EL219 significantly prolonged median survival in a dose-dependent manner compared to placebo (8, 10, and 16 days for the 1.5, 7.5, and 30 mg/kg doses, respectively, vs 7 days for placebo). Notably, EL219 at 7.5 and 30 mg/kg, as well as LAMB, improved overall survival compared to placebo (20%, 50%, and 10% survival for EL219 at 7.5 and 30 mg/kg, and LAMB, respectively, vs 0% for placebo). Importantly, EL219 at 30 mg/kg demonstrated superior efficacy over LAMB, with a median survival of 16 days and 50% overall survival, compared to 8.5 days and 10% survival for LAMB (*P* = 0.0273). EL219 at this dose showed no observable toxicity in the immunosuppressed mouse model of IPA (Fig. 1).

**Fig 2:**
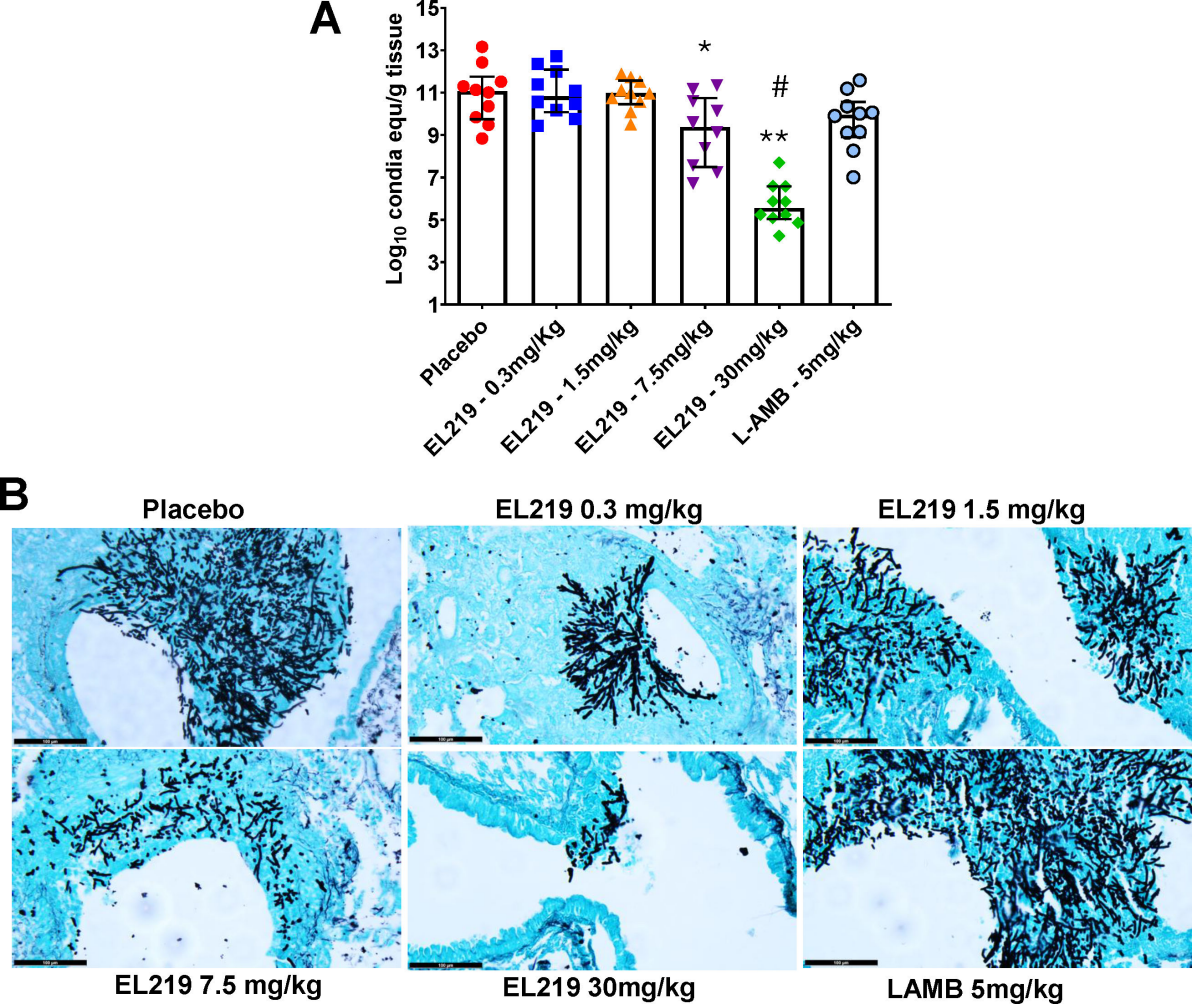
Reduction in lungs tissue fungal burden and histological examination of lung sections of immunosuppressed mice infected with *A. fumigatus*. (**A**) Mice (*n* = 10/group) infected with *A. fumigatus* via inhalation and treated daily with EL219 or LAMB were euthanized on Day +4 post-infection ~6 h after last treatment. EL219 at 7.5 or 30 mg/kg resulted in 1.5-log and 5-log reduction, respectively. * or ** *P* = 0.04 and <0.0001 versus placebo, respectively, and # *P* <0.0001 = versus LAMB. (**B**) Lung sections stained with GMS show that mice treated with EL219 had reduced fungal abscesses with shorter damaged hyphae in the lung tissues versus placebo-treated or LAMB-treated mice. Black bar is 100 µm.

Because EL219 treatment improved survival of mice infected with *A. fumigatus* over placebo treatment, we evaluated the effects of the drug on the tissue fungal burden in lungs, the primary target organ (24). Mice were infected and treated as above, and ~6 h after the last treatment, mice were humanely euthanized on Day 4 post-inoculation, and lungs were harvested and processed for tissue fungal burden determination as measured by the log_10_ conidia equivalents (CE) per gram of tissue using quantitative PCR (qPCR) (25). EL219 7.5 and 30 mg/kg treatments resulted in significant reductions of ~1.5-log and ~5-log, respectively, compared to the lung fungal burden in placebo-treated mice. However, none of the lower doses of EL219 (0.3 and 1.5 mg/kg) had an effect on the lung fungal burden. LAMB (5 mg/kg) trended to lower lung fungal burden by ~1.0-log compared to placebo-treated mice (*P* = 0.1) (Fig. 2A).

To further validate these findings, histopathological analysis was performed on lung tissues collected from the same animals. As expected, mice treated with EL219 at 1.5 and 7.5 mg/kg showed less dense fungal abscesses and shorter hyphae compared to placebo-treated mice. Remarkably, lungs from mice treated with 30 mg/kg EL219 displayed sporadic, severely damaged hyphae, indicating potent antifungal activity. In contrast, lungs from mice treated with EL219 at 0.3 mg/kg or LAMB showed only marginal improvements in tissue architecture compared to placebo (Fig. 2B).

Collectively, these results confirm that EL219—particularly at 30 mg/kg, and to a lesser extent at 7.5 mg/kg—is effective in improving survival, reducing fungal burden, and preserving lung tissue architecture in a murine model of IPA due to *A. fumigatus* infection.

### EL219 protects immunosuppressed mice from IPA due to *A. lentulus* and *A. calidoustus*

**Fig 3:**
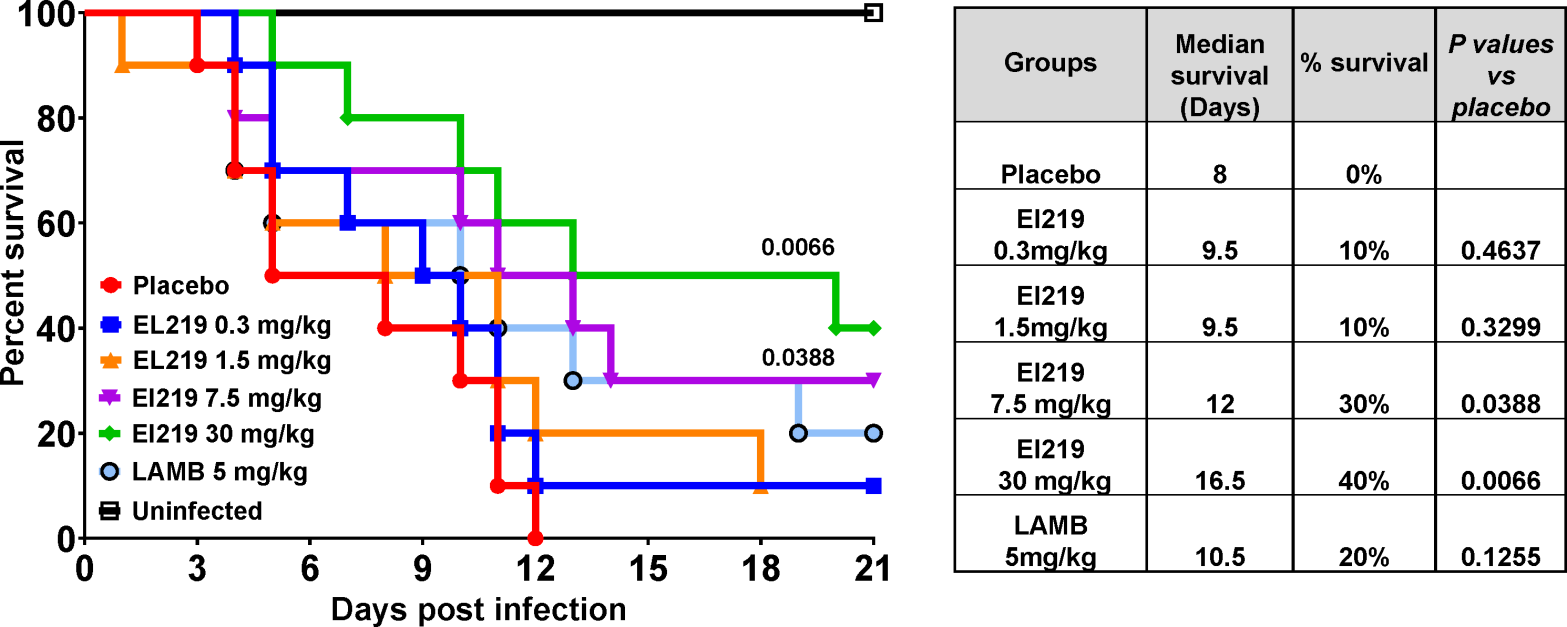
Survival of immunosuppressed mice infected with *A. lentulus* (Al-1) and treated with varying doses of EL219. Immunosuppressed mice (*n* = 10/group) were infected via intratracheal instillation with a verified inoculum of 1.0×10^4^ conidia delivered to the lungs. Treatment was administered once daily for four consecutive days, starting 16 h post-infection. *P* values shown on the graph represent statistical comparisons versus placebo-treated mice. The accompanying table summarizes median survival times and overall survival percentages for each treatment group.

To evaluate the broad-spectrum potential of EL219, we extended its *in vivo* efficacy testing to drug-resistant *A. lentulus* (Al-1) and *A. calidoustus* (Ac-6) in immunosuppressed mouse models of invasive aspergillosis. Initially, two independent pilot survival experiments were conducted to establish intratracheal infection models for each strain using varying inoculum concentrations (Fig. S1 and S2). Based on these studies, an inoculum of 2.5×10^6^ conidia per mouse was selected for both species to reliably induce infection. Subsequently, survival studies were performed to assess the therapeutic efficacy of EL219 at doses of 0.3, 1.5, 5.0, and 30 mg/kg, compared to LAMB at 5 mg/kg, in immunosuppressed mice infected with either *A. lentulus* or *A. calidoustus*. In the *A. lentulus* (Al-1) model, EL219 at 7.5 and 30 mg/kg significantly improved survival over placebo-treated mice (30% and 40% for EL219 at 7.5 and 30 mg/kg vs 0% for placebo, respectively). EL219 at 7.5 and 30 mg/kg also prolonged median survival times with 12 and 16.5 days, respectively, versus 8 days for placebo. In contrast, LAMB at 5 mg/kg achieved 20% survival, which was not significantly better than placebo-treated mice (*P* = 0.125) (Fig. 3).

**Fig 4:**
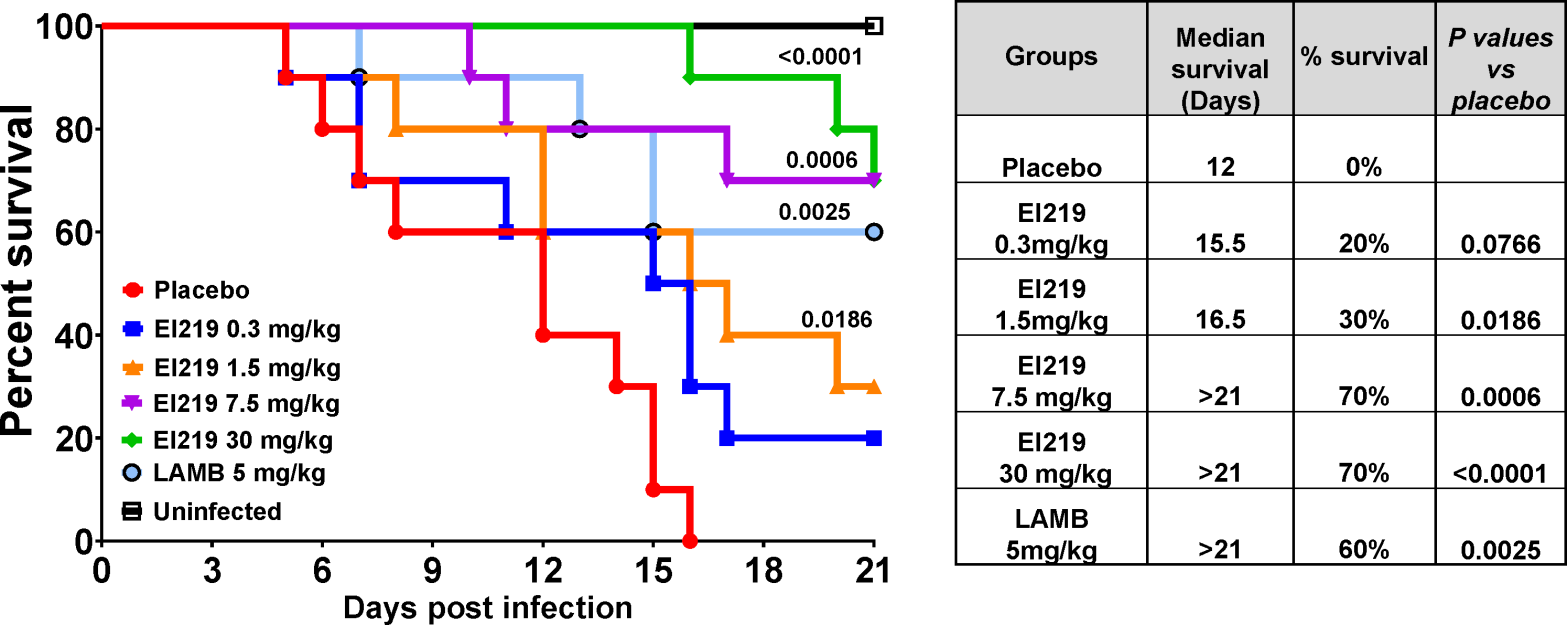
Survival of immunosuppressed mice infected with *A. calidoustus* (Ac-6) and treated with different doses of EL219. Immunosuppressed mice (*n* = 10/group) were infected via intratracheal instillation with a verified inoculum of 2.8×10^4^ conidia per mouse. Treatment was initiated 16 h post-infection and administered once daily for seven consecutive days. *P* values shown on the graph indicate statistical comparisons versus placebo-treated mice. The accompanying table presents median survival times and overall survival percentages through Day 21 post-infection.

In the *A. calidoustus* (Ac-6) model, EL219 at 1.5, 7.5, and 30 mg/kg, as well as LAMB at 5 mg/kg, significantly increased survival of mice compared to placebo (30%, 70%, 70%, and 60% vs 0%, respectively). Notably, EL219 at 7.5 and 30 mg/kg and LAMB extended median survival to beyond 21 days, while placebo-treated mice had a median survival of 12 days (Fig. 4). Importantly, no observable toxicity was associated with EL219 at 30 mg/kg in either infection model. These results highlight the extended efficacy and safety of EL219 against azole-resistant and amphotericin B-resistant *Aspergillus* species and support its potential use as a broad-spectrum antifungal agent.

## DISCUSSION

IPA continues to pose a significant clinical challenge, especially in immunocompromised individuals such as those undergoing chemotherapy or transplantation. Although *A. fumigatus* remains the predominant species responsible for IPA, emerging cryptic species like *A. lentulus* and *A. calidoustus* are increasingly reported (4, 7). These pathogens not only may exhibit reduced susceptibility or real resistance to multiple antifungal agents, including amphotericin B and triazoles, but are also associated with higher mortality rates and diagnostic difficulties (11, 12). The limited efficacy and toxicity concerns of current therapies underscore the urgent need for safer and more broadly effective antifungal agents (15).

Our study demonstrates that EL219, a rationally engineered next-generation polyene, exhibits potent *in vitro* activity and broad-spectrum *in vivo* efficacy against multiple clinically relevant *Aspergillus* species. In murine models of IPA caused by *A. fumigatus*, EL219 showed dose-dependent improvements in survival and substantial reductions in lung fungal burden. Furthermore, the histopathological analysis further confirmed that high-dose EL219 treatment was associated with reduced fungal tissue invasion and preservation of lung architecture, consistent with microbiological and survival outcomes. The ability of EL219 to enhance the overall survival of immunosuppressed mice was extended to those infected with the drug-resistant *A. lentulus* and *A. calidoustus*. Notably, EL219 at 30 mg/kg was well tolerated and outperformed LAMB at 5 mg/kg, a dose in mice that achieves exposures that are similar to or exceed the exposures achieved by the recommended dose of 3–5 mg/kg/day for treating patients with IPA (26, 27), even against isolates exhibiting resistance to conventional polyenes. These findings are particularly important given the growing incidence of infections with antifungal-resistant *Aspergillus* spp. other than *fumigatus* that commonly fail current therapy options.

Despite these encouraging results, our study has several limitations. First, although we included representative clinical isolates of *A. fumigatus*, *A. lentulus*, and *A. calidoustus*, the number of strains tested *in vivo* was limited and it is possible that outcomes may differ with other strains of each species tested. A broader panel of genetically and geographically diverse isolates would be necessary to fully characterize EL219’s activity spectrum. Second, while the study demonstrated survival and fungal burden outcomes, we did not investigate detailed pharmacokinetic/pharmacodynamic relationships, which will be critical for clinical translation (22). Despite these limitations, EL219 shows promise as a novel, broad-spectrum polyene with superior activity to LAMB, particularly against resistant *Aspergillus* species, warranting further investigations and eventually translating into clinical trial testing against IPA.

## MATERIALS AND METHODS

### Isolates and culture conditions

*A. fumigatus* Af293 was isolated from lung tissue taken at autopsy from a patient who had developed a low white cell count after gold therapy for rheumatoid arthritis*.* Other *A. fumigatus* isolates (Af-1, Af-2, Af-3, Af-4, and Af-5), *A. lentulus* isolates (Al-1, Al-2, Al-3, Al-4, Al-5, and Al-6), and *A. calidoustus* (Ac-1, Ac-2, Ac-3, Ac-4, Ac-5, and Ac-6*)* were provided by the Fungus Testing Laboratory, University of Texas Health Science Center at San Antonio. The organisms were grown on Sabouraud dextrose agar for 5–7 days at 37°C. Conidia were collected in endotoxin-free PBS containing 0.01% Tween 80, washed with PBS, and then counted with a hemocytometer to prepare the final concentration.

### Susceptibility testing

The *in vitro* susceptibility of EL219 (Elion Therapeutics, Inc., Boston, MA, USA), LAMB (Gilead Sciences, Inc., Foster City, CA, USA), or amphotericin B (AMB; Sigma-Aldrich, Burlington, MA, USA) against *A. fumigatus*, *A. lentulus*, or *A. calidoustus* isolates was assessed using the CLSI M38 broth microdilution method (23). EL219 and LAMB were reconstituted to yield final working concentrations following the manufacturer’s instructions, and AMB stocks were prepared in DMSO with further dilutions in RPMI 1640 buffered with MOPS (0.165 M, pH 7.0). The concentration range of each agent tested was 0.03–16 mg/mL. MICs for EL219, AMB, and LAMB were visually assessed at 100% growth inhibition compared to the drug-free control after 48 h of incubation at 35°C.

### Immunosuppression

Male CD-1 mice (23–28 g; Envigo, Indianapolis, IN, USA) were used for this study. Neutropenia was induced by administering cyclophosphamide (200 mg/kg, intraperitoneally) and cortisone acetate (500 mg/kg, subcutaneously) on Days −2 and +3, relative to infection. This immunosuppressive regimen results in approximately 14 days of leukopenia, with total white blood cell counts decreasing from ~130,000/cm^3^ to nearly undetectable levels, as measured using the Unopette System (Becton-Dickinson and Co.) (28). To prevent bacterial infection, 50 mg/L enrofloxacin (Baytril; Bayer) was added to drinking water on Day −3, then switched to daily ceftazidime treatment (5 mg/mouse, subcutaneous injection) starting on the day of infection (Day 0) through Day 13 (29).

### Infection

Immunosuppressed mice were challenged with *A. fumigatus* Af293 in an inhalation chamber by aerosolizing 12 mL of a 1×10^9^/mL suspension of conidia with a small particle nebulizer driven by compressed air (24). A standard exposure time of one hour was used for all experiments. Immediately after infection, a subset of mice (*n* = 3) was sacrificed. Their lungs were removed, homogenized, and quantitatively cultured to determine the number of conidia delivered to the mouse. This exposure reproducibly delivers 1–5×10^3^ conidia per mouse and produces infection in 100% of animals with 70–100% mortality by 2 weeks.

For *A. lentulus* (Al-1) and *A. calidoustus* (Ac-6), we established the infection model by intratracheal instillation (28). Immunosuppressed mice were challenged with varying inocula (2.5×10^4^, 2.5×10^5^, and 2.5×10^6^ conidia) in a 25 µL volume per mouse following sedation with isoflurane gas. A pilot experiment was conducted to optimize and determine the effective infectious dose (*n* = 5/group). Based on the results, 2.5×10^6^ conidia per strain was selected for subsequent efficacy studies. In all experiments, immediately post-infection, a subset of mice (*n* = 3) was sacrificed, and lungs were harvested and quantitatively cultured to confirm the delivered inoculum.

### Treatment

Treatment with placebo (diluent control 5% DW), EL219 (0.3 , 1.5, 7.5 , or 30 mg/kg once daily [QD]), or LAMB (5 mg/kg, QD), began 16 h post-infection and continued for four consecutive days for *A. fumigatus* and *A. lentulus* and 7 days for *A. calidoustus* given by intravenous injection for both drugs. Dose selection was driven by our past experience with EL219 (19) and by comparable dosing of LAMB clinically (3, 27). The primary endpoint was time to moribundity (survival over 21 days), while the secondary endpoint was fungal burden in lung tissue for *A. fumigatus*. Fungal burden was quantified by measuring CE per gram of lung tissue using qPCR (25). In addition, lung sections from representative mice in each treatment group (*n* = 2/group) were examined histologically using Grocott’s methenamine silver (GMS) staining for microscopic evaluation. Control groups of uninfected, immunosuppressed mice were included in the survival studies.

### Statistical analysis

For survival studies, based on the vast experience with animal models, it was expected that 10 mice/group would provide at least 80% power to test the hazard ratio of 0.2 or less, with a level of significance *P* = 0.025 using the Cox proportional hazard model (one-sided test), assuming 100% and 50% mortality in the test and control group, respectively. For the tissue pathogen burden, 10 mice/group would provide approximately 90% statistical power to detect the effect size of 2.5 or 2.5 SD difference in CFU (expressed as log) content using a two-sided two-sample *t*-test with an α of 0.05, assuming the standard deviation of the test group is twice that of the control group. When multiple experimental conditions were compared, the one-way ANOVA with Tukey’s multiple comparison test was used. For all comparisons, mean ± SD, median (interquartile range), and 95% CI were computed, and *P* values of <0.05 were considered significant. All data analyses were conducted using GraphPad Prism 6.

## SUPPLEMENTAL MATERIAL

Supplemental material aac.01400-25-s0001.docx Fig. S1 and S2.

ASM does not own the copyrights to Supplemental Material that may be linked to, or accessed through, an article. The authors have granted ASM a non-exclusive, world-wide license to publish the Supplemental Material files. Please contact the corresponding author directly for reuse.

## References

[1] Marr KA, Carter RA, Crippa F, Wald A, Corey L. 2002. Epidemiology and outcome of mould infections in hematopoietic stem cell transplant recipients. Clin Infect Dis 34:909–917. doi:10.1086/339202 11880955

[2] Kontoyiannis DP, Bodey GP. 2002. Invasive aspergillosis in 2002: an update. Eur J Clin Microbiol Infect Dis 21:161–172. doi:10.1007/s10096-002-0699-z 11957017

[3] Lamoth F, Calandra T. 2022. Pulmonary aspergillosis: diagnosis and treatment. Eur Respir Rev 31:220114. doi:10.1183/16000617.0114-2022 36450372 PMC9724826

[4] Gaffney S, Kelly DM, Rameli PM, Kelleher E, Martin-Loeches I. 2023. Invasive pulmonary aspergillosis in the intensive care unit: current challenges and best practices. APMIS 131:654–667. doi:10.1111/apm.13316 37022291

[5] Denning DW, Cadranel J, Beigelman-Aubry C, Ader F, Chakrabarti A, Blot S, Ullmann AJ, Dimopoulos G, Lange C. 2016. Chronic pulmonary aspergillosis: rationale and clinical guidelines for diagnosis and management. Eur Respir J 47:45–68. doi:10.1183/13993003.00583-2015 26699723

[6] Dagenais TRT, Keller NP. 2009. Pathogenesis of Aspergillus fumigatus in invasive aspergillosis. Clin Microbiol Rev 22:447–465. doi:10.1128/CMR.00055-08 19597008 PMC2708386

[7] Kanaujia R, Singh S, Rudramurthy SM. 2023. Aspergillosis: an update on clinical spectrum, diagnostic schemes, and management. Curr Fungal Infect Rep 4:1–12. doi:10.1007/s12281-023-00461-5 PMC10157594 37360858

[8] Djenontin E, Lavergne RA, Morio F, Dannaoui E. 2025. Antifungal resistance in non-fumigatus Aspergillus species. Mycoses 68:e70051. doi:10.1111/myc.70051 40219727 PMC11992613

[9] Lamoth F. 2016. Aspergillus fumigatus-related species in clinical practice. Front Microbiol 7:683. doi:10.3389/fmicb.2016.00683 27242710 PMC4868848

[10] Negri CE, Gonçalves SS, Sousa ACP, Bergamasco MD, Martino MDV, Queiroz-Telles F, Aquino VR, Castro P de TO, Hagen F, Meis JF, Colombo AL. 2017. Triazole resistance is still not emerging in Aspergillus fumigatus isolates causing invasive aspergillosis in brazilian patients. Antimicrob Agents Chemother 61:00608–00617. doi:10.1128/AAC.00608-17 PMC5655068 28893772

[11] Bosetti D, Neofytos D. 2023. Invasive aspergillosis and the impact of azole-resistance. Curr Fungal Infect Rep:1–10. doi:10.1007/s12281-023-00459-z PMC10024029 37360857

[12] Cheng J, Han H, Kang W, Cai Z, Zhan P, Lv T. 2024. Comparison of antifungal drugs in the treatment of invasive pulmonary aspergillosis: a systematic review and network meta-analysis. Front Microbiol 15:1504826. doi:10.3389/fmicb.2024.1504826 39687872 PMC11648418

[13] Boyer J, Feys S, Zsifkovits I, Hoenigl M, Egger M. 2023. Treatment of invasive aspergillosis: how it’s going, where it’s heading. Mycopathologia 188:667–681. doi:10.1007/s11046-023-00727-z 37100963 PMC10132806

[14] Ritter J. 2002. Amphotericin B and its lipid formulations. Mycoses 45 Suppl 3:34–38. doi:10.1111/j.1439-0507.2002.tb04767.x 12690969

[15] Stone NRH, Bicanic T, Salim R, Hope W. 2016. Liposomal amphotericin B (AmBisome): a review of the pharmacokinetics, pharmacodynamics, clinical experience and future directions. Drugs (Abingdon Engl) 76:485–500. doi:10.1007/s40265-016-0538-7 PMC4856207 26818726

[16] Meis JF, Chowdhary A, Rhodes JL, Fisher MC, Verweij PE. 2016. Clinical implications of globally emerging azole resistance in Aspergillus fumigatus. Philos Trans R Soc Lond B Biol Sci 371:20150460. doi:10.1098/rstb.2015.0460 28080986 PMC5095539

[17] Kousha M, Tadi R, Soubani AO. 2011. Pulmonary aspergillosis: a clinical review. Eur Respir Rev 20:156–174. doi:10.1183/09059180.00001011 21881144 PMC9584108

[18] Mousset S, Buchheidt D, Heinz W, Ruhnke M, Cornely OA, Egerer G, Krüger W, Link H, Neumann S, Ostermann H, Panse J, Penack O, Rieger C, Schmidt-Hieber M, Silling G, Südhoff T, Ullmann AJ, Wolf HH, Maschmeyer G, Böhme A. 2014. Treatment of invasive fungal infections in cancer patients-updated recommendations of the Infectious Diseases Working Party (AGIHO) of the German Society of Hematology and Oncology (DGHO). Ann Hematol 93:13–32. doi:10.1007/s00277-013-1867-1 24026426 PMC3889633

[19] Maji A, Soutar CP, Zhang J, Lewandowska A, Uno BE, Yan S, Shelke Y, Murhade G, Nimerovsky E, Borcik CG, et al.. 2023. Tuning sterol extraction kinetics yields a renal-sparing polyene antifungal. Nature 623:1079–1085. doi:10.1038/s41586-023-06710-4 37938782 PMC10883201

[20] Vahedi-Shahandashti R, Lass-Flörl C. 2025. In vitro activity of SF001: a next-generation polyene versus amphotericin B. Antimicrob Agents Chemother 69:e0032225. doi:10.1128/aac.00322-25 40261080 PMC12135503

[21] Gebremariam T, Gu Y, Patterson H, Youssef E, Alkhazraji S, Elsayed T, Wiederhold NP, Ibrahim AS. 2025. Efficacy assessments of SF001, a next-generation polyene antifungal, in a neutropenic mouse model of invasive fusariosis. Antimicrob Agents Chemother 69:e0180224. doi:10.1128/aac.01802-24 40167370 PMC12057364

[22] Lepak AJ, VanScoy B, Rubino C, Ambrose PG, Andes DR. 2024. In vivo pharmacodynamic characterization of a next-generation polyene, SF001, in the invasive pulmonary aspergillosis mouse model. Antimicrob Agents Chemother 68:e0163123. doi:10.1128/aac.01631-23 38319077 PMC10916380

[23] Institute. CLS. 2008. Reference Method for Broth Dilution Antifungal Susceptibility Testing of Filamentous Fungi M38-A2. CLSI, PA, USA.

[24] Sheppard DC, Rieg G, Chiang LY, Filler SG, Edwards JE, Ibrahim AS. 2004. Novel inhalational murine model of invasive pulmonary aspergillosis. Antimicrob Agents Chemother 48:1908–1911. doi:10.1128/AAC.48.5.1908-1911.2004 15105158 PMC400587

[25] Bowman JC, Abruzzo GK, Anderson JW, Flattery AM, Gill CJ, Pikounis VB, Schmatz DM, Liberator PA, Douglas CM. 2001. Quantitative PCR assay to measure Aspergillus fumigatus burden in a murine model of disseminated aspergillosis: demonstration of efficacy of caspofungin acetate. Antimicrob Agents Chemother 45:3474–3481. doi:10.1128/AAC.45.12.3474-3481.2001 11709327 PMC90856

[26] Douglas AP, Smibert OC, Bajel A, Halliday CL, Lavee O, McMullan B, Yong MK, van Hal SJ, Chen SC-A, Australasian Antifungal Guidelines Steering Committee. 2021. Consensus guidelines for the diagnosis and management of invasive aspergillosis, 2021. Intern Med J 51 Suppl 7:143–176. doi:10.1111/imj.15591 34937136

[27] Patterson TF, Thompson GR III, Denning DW, Fishman JA, Hadley S, Herbrecht R, Kontoyiannis DP, Marr KA, Morrison VA, Nguyen MH, Segal BH, Steinbach WJ, Stevens DA, Walsh TJ, Wingard JR, Young J-AH, Bennett JE. 2016. Practice guidelines for the diagnosis and management of aspergillosis: 2016 update by the Infectious Diseases Society of America. Clin Infect Dis 63:e1–e60. doi:10.1093/cid/ciw326 27365388 PMC4967602

[28] Luo G, Gebremariam T, Lee H, French SW, Wiederhold NP, Patterson TF, Filler SG, Ibrahim AS. 2013. Efficacy of liposomal amphotericin B and posaconazole in intratracheal models of murine mucormycosis. Antimicrob Agents Chemother 57:3340–3347. doi:10.1128/AAC.00313-13 23650163 PMC3697351

[29] Gebremariam T, Alkhazraji S, Alqarihi A, Wiederhold NP, Shaw KJ, Patterson TF, Filler SG, Ibrahim AS. 2020. Fosmanogepix (APX001) is effective in the treatment of pulmonary murine mucormycosis due to rhizopus arrhizus. Antimicrob Agents and Chemother. doi:10.1128/aac.00178-20:AAC.00178-20 PMC7269494 32205345

