# Supplementary material for: Efficacy assessments of EL219, a next-generation polyene antifungal, in immunosuppressed mice infected with drug-sensitive and drug-resistant Aspergillus isolates
Source: Antimicrob Agents Chemother. 2025 Dec 5;70(1):e01400-25. doi: 10.1128/aac.01400-25 (PMC12777570; doi:10.1128/aac.01400-25)
Supplement: Supplemental material — Fig. S1 and S2. [file aac.01400-25-s0001.docx]

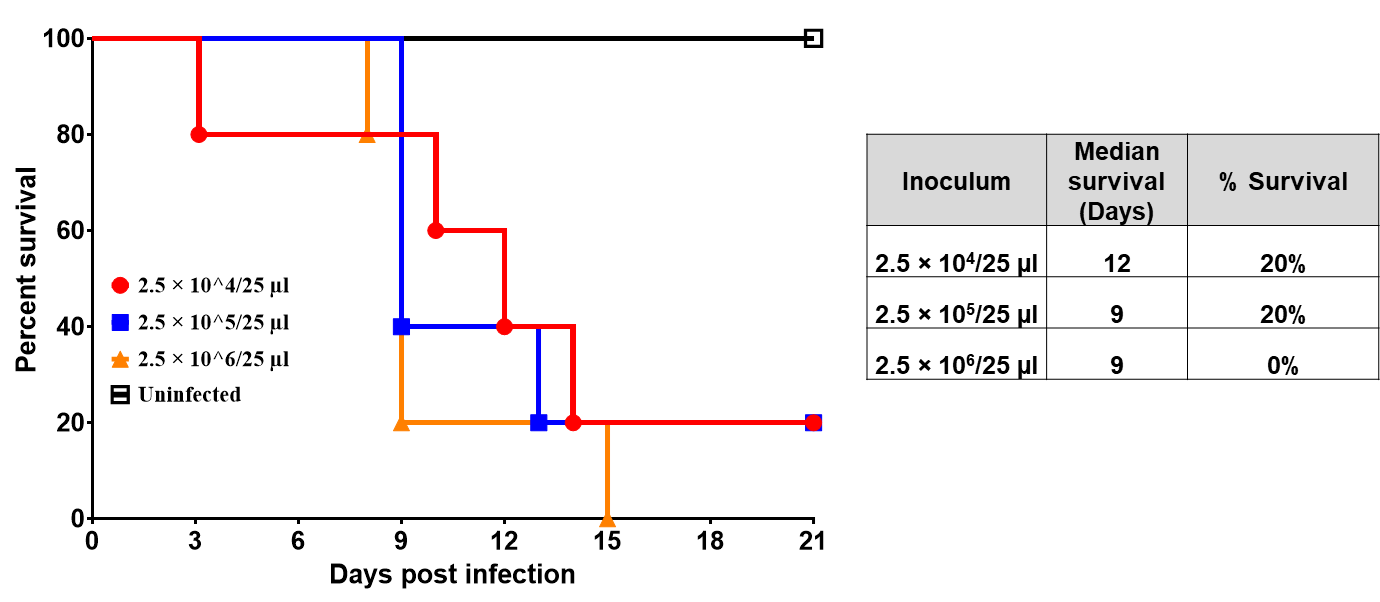


**Fig S1. Survival of immunosuppressed mice infected with different inoculum of *A. lentulus*.** Data in the Table include the median survival times and the overall survival by day 21 post infection.


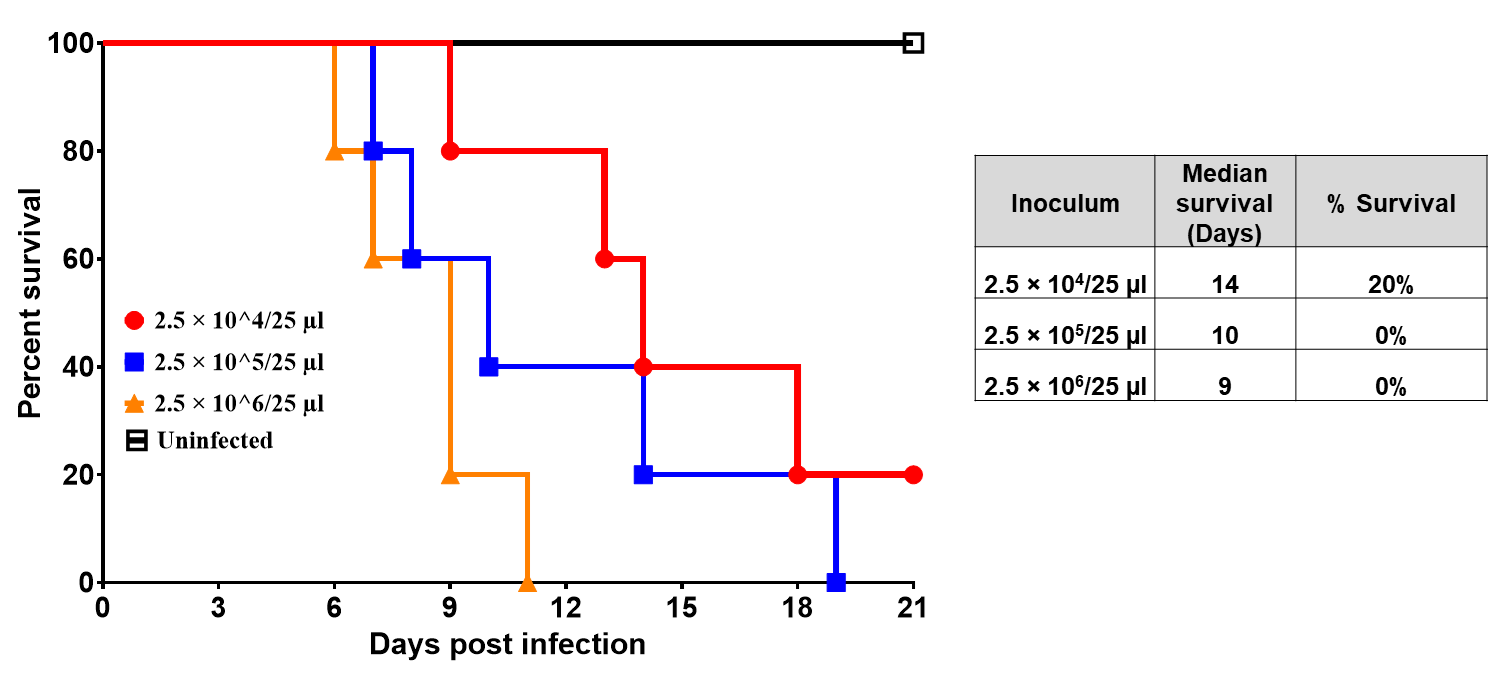


**Fig S2. Survival of immunosuppressed mice infected with different inoculum of *A. calidoustus*.** Data in the Table include the median survival times and the overall survival by 21 post infection.
